# Possible non-sylvatic transmission of yellow fever between non-human primates in São Paulo city, Brazil, 2017–2018

**DOI:** 10.1038/s41598-020-72794-x

**Published:** 2020-09-25

**Authors:** Mariana Sequetin Cunha, Rosa Maria Tubaki, Regiane Maria Tironi de Menezes, Mariza Pereira, Giovana Santos Caleiro, Esmenia Coelho, Leila del Castillo Saad, Natalia Coelho Couto de Azevedo Fernandes, Juliana Mariotti Guerra, Juliana Silva Nogueira, Juliana Laurito Summa, Amanda Aparecida Cardoso Coimbra, Ticiana Zwarg, Steven S. Witkin, Luís Filipe Mucci, Maria do Carmo Sampaio Tavares Timenetsky, Ester Cerdeira Sabino, Juliana Telles de Deus

**Affiliations:** 1 Vector-Borne Diseases Laboratory, Adolfo Lutz Institute, Sao Paulo, Brazil; 2 Laboratory of Medical Entomology, Superintendence of Control of Endemic Diseases (SUCEN), Sao Paulo, Brazil; 3 Yellow Fever Technical Group, Superintendence of Control of Endemic Diseases (SUCEN), Sao Paulo, Brazil; 4 Laboratory of Virology, Institute of Tropical Medicine, University of São Paulo, Sao Paulo, Brazil; 5 Divisao de Zoonoses, Centro de Vigilância Epidemiológica, Sao Paulo, Brazil; 6 Pathology Center, Adolfo Lutz Institute, Sao Paulo, Brazil; 7 Technical Division of Veterinary Medicine and Wildlife, Sao Paulo, Brazil; 8 Department of Obstetrics and Gynecology, Weill Cornell Medicine, New York, NY 10065 USA; 9 Center of Virology, Adolfo Lutz Institute, Sao Paulo, Brazil

**Keywords:** Virology, Viral epidemiology

## Abstract

Yellow Fever (YF) is a severe disease caused by Yellow Fever Virus (YFV), endemic in some parts of Africa and America. In Brazil, YFV is maintained by a sylvatic transmission cycle involving non-human primates (NHP) and forest canopy-dwelling mosquitoes, mainly *Haemagogus*-spp and *Sabethes-*spp. Beginning in 2016, Brazil faced one of the largest Yellow Fever (YF) outbreaks in recent decades, mainly in the southeastern region. In São Paulo city, YFV was detected in October 2017 in *Aloutta* monkeys in an Atlantic Forest area. From 542 NHP, a total of 162 NHP were YFV positive by RT-qPCR and/or immunohistochemistry, being 22 *Callithrix-*spp. most from urban areas. Entomological collections executed did not detect the presence of strictly sylvatic mosquitoes. Three mosquito pools were positive for YFV, 2 *Haemagogus leucocelaenus*, and 1 *Aedes scapularis*. In summary, YFV in the São Paulo urban area was detected mainly in resident marmosets, and synanthropic mosquitoes were likely involved in viral transmission.

## Introduction

Yellow Fever virus (YFV) is an arbovirus member of the *Flavivirus* genus, family *Flavivirida*e and the causative agent of yellow fever (YF)^1^. The disease is considered endemic in parts of South America and Africa, with 200,000 cases and 30,000 deaths annually^2^. In Africa there are three transmission cycles: the sylvatic one, involving NHP and sylvatic *Aedes africanus*, the intermediate cycle, involving peridomestic *Aedes* spp, and the urban cycle, which involves mainly *Aedes aegypti*^3,4^. In Brazil, only the sylvatic YFV cycle is now described, subsequent to the eradication of *Aedes aegypti* and urban cycle transmission in 1942. Occasional spillover to non-vaccinated humans occur when they encroach into forested areas^4^. The current transmission cycle involves the mosquito vectors *Haemagogus* sp. and *Sabethes* sp., and several species of non-human primates (NHP), including the genera *Alouatta*, *Sapajus*, *Callicebus* and *Callithrix*^5^ . Most New World NHPs are susceptible to YFV, especially individuals of the *Alouatta* genus. The Brazilian surveillance system is based upon YFV detection in dead NHP^6^. Its identification triggers mass vaccination in local populations^4^.

In Brazil, during the late twentieth century and extending to the first decade of the twenty first century, intense YFV circulation extended from the Amazon region to the contiguous states of Central Brazil^7^. Subsequently, starting in 2016, several YFV outbreaks were reported in states in the most populous southeastern region, caused by a new YFV belonging to South American lineage I that was introduced from the Midwest region and disseminated to areas that were considered free of yellow fever^8^. This resulted in the most severe outbreak in past decades in the southeastern region, comprising the states of São Paulo, Rio de Janeiro, Minas Gerais and Espírito Santo. During the 2017–2018 outbreak, São Paulo State had 3,352 human cases with 333 fatalities, and a total of 534 YF epizootic events^9,10^. However, despite the magnitude of this outbreak, little is known about which mosquito species were involved in some of these YFV transmission areas.

We now present the results of YF confirmed epizootic events and entomological investigations conducted in Sao Paulo city during the intense circulation of YFV between 2017 and 2018. Particular attention is paid to urban and urban green areas, where no vectors classically involved in the sylvatic YFV transmission were found. Our objective was to describe YFV-positive epizootic events that were probably associated with *Aedes* spp. mosquitoes instead of the *Haemagogus* spp or *Sabethes* spp primary and secondary vectors.

## Material and methods

### Study area

This is a descriptive study regarding positive YFV NHP and mosquitoes collected in the city of Sao Paulo, which is the capital of Sao Paulo State, located in the Southeast Region of Brazil. It is the largest city in the country, with about 12 million inhabitants in an area of 1,521.11 km^2^ divided into 96 districts. São Paulo has a subtropical humid climate, with a rainy season during the summer (December-March) and a dry season during the autumn/winter (April-August).

### Non-human primates sampling and diagnosis

From October 2017 to December, 2018, carcasses of NHP were collected by local authorities and later identified and autopsied by the veterinary staff from *Divisão Técnica de Medicina Veterinária e Manejo da Fauna Silvestre* (DEPAVE-3), and results reported to the surveillance system (*Centro de Vigilância Epidemiológica*—CVE). Samples of liver, brain and spleen were sent to Adolfo Lutz Institute, the reference laboratory for YFV, according to Ministry of Health guidelines^6^. Liver and brain samples were lysed using Magna Lyser green beads (Roche Life Sciences, Penzberg, Germany) and extracted using RNeasy Mini Kit (Qiagen, Hilden, Germany) according to the manufacturer’s instructions. YFV diagnosis was made by RT-qPCR^11^, (cut-off value = 38 Cycle threshold) (CT), and immunohistochemistry (IHC). For IHC analysis, liver tissue sections were tested with an in house primary polyclonal anti-YF antibody (1:40.000), signal amplification was achieved with Super HighDef (Enzo Life Sciences, Farmingdale) and visualization was done with diaminobenzidine (D-5637; Sigma, St. Louis, MO). Positive results at IHC were considered when there was immunolabeling in the cytoplasm of hepatocytes, with brown or red precipitates. NHP localization was based on the address where they were collected. Information regarding NHP was filled in the *Sistema de Informação de Agravos de Notificação* (SINAN) form, according to the Ministry of Health. When not available, coordinates were obtained using Google Earth Pro.

### Mosquitoes sampling and YFV detection

Mosquitoes were collected by Sucen (Superintendence for Control of Endemic Diseases, State of São Paulo) at sites where epizootic events were recorded. They were captured at ground level between 9 am and 3 pm, by using an entomologic net and bottle-type manual vacuums in woods and green areas, and Nasci’s Aspirator in urban dwellings. After collection, they were frozen, transferred to cryogenic tubes, placed in liquid nitrogen containers for transportation to the laboratory and stored at -70 °C until processing. They were identified and separated according to species, place and date of collection into pools containing between 1–10 mosquitoes. Pools were triturated using FastPrep-24 5G Instrument (MP Biomedicals, OH) and magnetic beads in a 1 mL phosphate-buffered saline solution with 0.75% bovine albumin, penicillin (100 units/mL) and streptomycin (100 µg/mL). The resultant suspension was centrifuged at 1800 × g for 15 min, and the supernatant was withdrawn and frozen at −70 °C. Samples were extracted using the QIAamp RNA Viral Mini Kit (QIAGEN, Hilden, Germany), according to manufacturer’s instructions followed by RT-qPCR^11^. In addition, attempts for viral isolation were performed as follows: after medium removal, 20µL of positive RT-qPCR mosquito pools were inoculated into cell tubes containing monolayer cultures of C6/36 (*Aedes albopictus* clone C6/36 ATCC CRL-1660) and Vero (Vero ATCC CCL-81 *Cercopithecus aethiops* kidney normal) cells. C6/36 culture tubes were incubated for nine days at 28 °C with L-15 medium containing 2% FBS, penicillin (100units/mL) and streptomycin (100 µg/mL), while Vero tubes were incubated for nine days at 37 °C and 5% CO_2_ with 199 medium containing 2% FBS, penicillin (100units/mL) and streptomycin (100 µg/mL), and checked daily for a cytopathic effect. IFA tests were performed using YFV monoclonal antibodies provided by the U.S. Centers for Disease Control and Prevention, according to a published procedure^12^. A total of three passages were performed. In order to determine whether a positive pool of mosquito had recently consumed human or non-human primate blood an RNase P protocol^13^, used as an internal control for RT-qPCR, was performed. Mosquito density by entomological surveillance site was calculated as the arithmetic mean of collections performed, analyzed by collection method, effort and hours in each site^14^.

### YFV from São Paulo phylogenetic analysis

Whole genome sequences from NHP and humans from São Paulo previously generated^15,16^ were aligned with YFV sequences from the 2016–2018 Brazilian outbreak using MAFFT (available at https://mafft.cbrc.jp/alignment/server/) and maximum likelihood (ML) phylogenetic analyses were conducted with a TN93 model with gamma variation^17^ (available at https://www.atgc-montpellier.fr/). Tree was edited using FigTree v.14.3 with a mid-point root. A YFV South American lineage II was used as an outer group (accession number MF004382).

## Ethical statement

All animal research was approved by the Institutional Animal Care and Use Committee (IACUC) of the Adolfo Lutz Institute, São Paulo. The Adolfo Lutz Central Institute (The Central Public Health Laboratory from the State of São Paulo), an organ linked to the Health Department of the state of São Paulo, is the official laboratory for the diagnosis of YFV in humans and primates. NHP samples were sent by local authorities in accordance with Brazilian Ministry of Health guidelines (https://vigilancia.saude.mg.gov.br/index.php/download/guia-de-epizootias-febre-amarela-2a-edicao-2017/#). The use of NHP samples for research was also approved by ICMBIO protocol number 65181 (https://www.icmbio.gov.br/sisbio/).

## Results

### YFV in Non-human primates from urbanized areas

**Figure 1:**
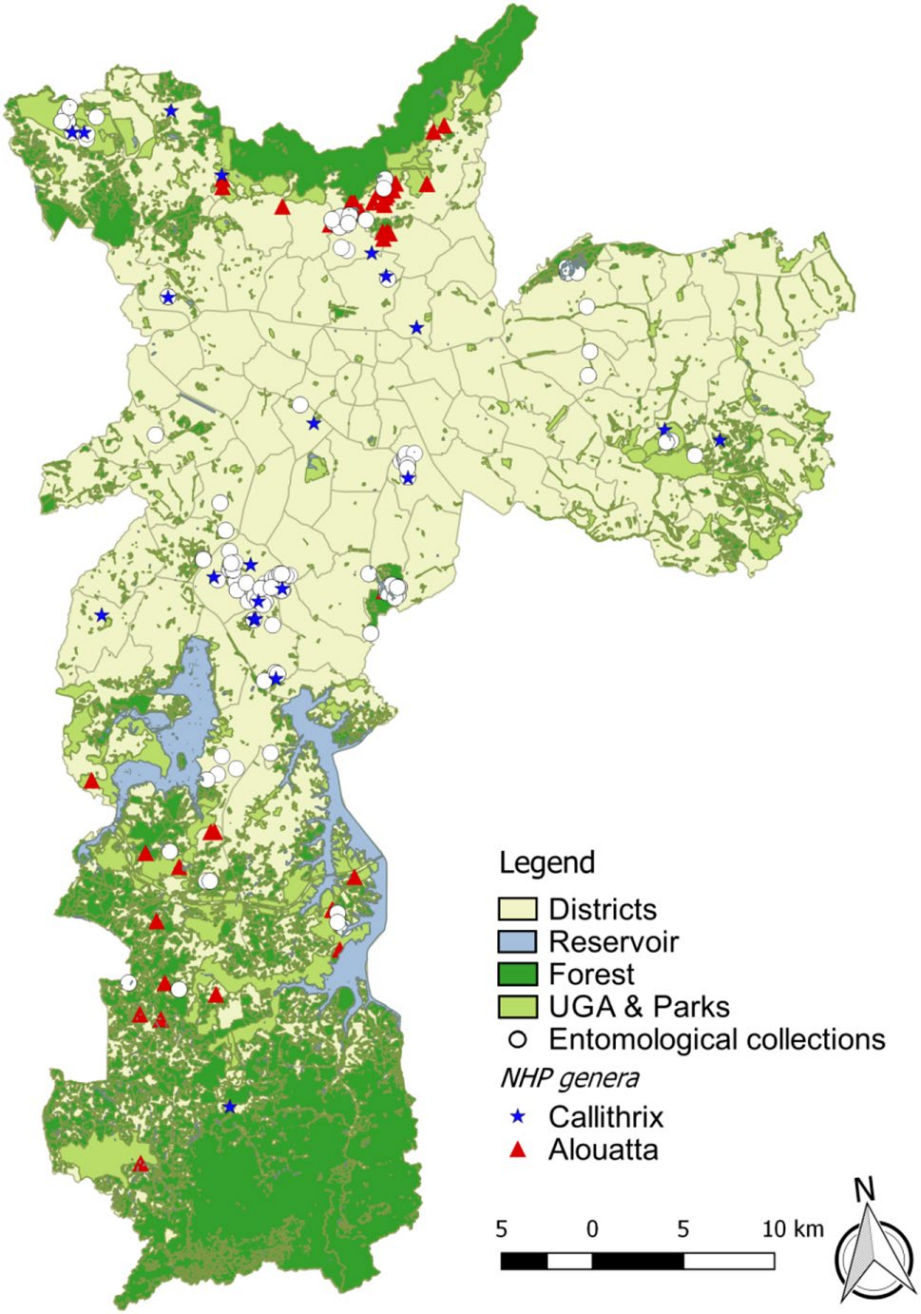
Map of São Paulo city with sites of Culicidae captures after confirmed epizootic events, 2017–2018. Districts represent the administrative divisions of the city. Reservoir represents the city water dams. Forest represents the Atlantic Forest protections area. Urban Green Areas (UGA) consists of urban parks and squares. Map was generated using QGIS version 2.14.9 ( available at https://www.qgis.org/pt_BR/site/).

After almost a one hundred year absence, YFV was detected in Sao Paulo city in October 2017, in *Alouatta guariba clamitans* monkeys from Horto Florestal, an Atlantic Forest protection area located in the North region of the city (Tremembé district). During the 2017–2018 outbreak, a total of 542 NHP were sent for YFV diagnosis (Table 1), of which 162 (30%) were positive (n = 140 *Alouatta guariba clamitans*, median CT = 11, min = 7, max = 38); and 22 *Callithrix* sp. (median CT = 36, min = 33, max = 38). All positive *Callithrix* monkeys were classified as hybrid individuals based on their morphology. Most positive NHP, mainly of the *Alouatta* genera, were found near forest areas located in the North and South zones (Green Belt of São Paulo City, GBSPC) (Fig. 1). From the 22 *Callithrix sp.,* 4 (18,1%) were collected in wooden areas (WO), 5 (22,8%) in urban green areas (UGA) and 13 (59,1%) in urban areas (UR). One *Callithrix* was a captive animal from the Mandaqui district (North Zone-animal ID 14), with no outdoor access according to its owner. A total of 18 animals were positive during the epidemic period (December-May), while 4 animals were positive during the non-epidemic period (June-November). The last confirmed epizootic event was in September 2018, and cases were detected throughout this period. Information regarding positive *Callithrix* is depicted in Table 2. Also, we found one positive free living *Alouatta guariba clamitans* monkey in the city Zoo on January 10th, 2018, which is located in a remnant Atlantic Forest area of the Cursino district. However, this location had no connection with sylvatic areas, and no epizootic events were confirmed nearby. The distribution of positive *Callithrix* and *Alouatta* NHP provides evidence for the restriction of the last monkey genera to woods and the dispersion of *Callithrix (*marmosets) throughout urbanized areas.

**Table 1:** Total of notified and confirmed epizootic events in São Paulo, SP, Brazil, 2017–2018.

NHP	2017		2018		Total	P(%)
	N (%)	P (%)	N (%)	P (%)		
*Aloutta g. clamitans*	141 (53.6)	95 (94)	68 (24.4)	45 (72.5)	209 (38.5)	140 (85.9)
*Callithrix sp.*	119 (45.2)	5 (6)	206 (73.8)	17 (27.4)	324 (60)	22 (14.1)
*Sapajus nigritus*	2 (0.8)	0	5 (1.8)	0	7 (1.3)	0
*Callicebus* *nigrifus*	1 (0.4)	0	0	0	1 (0.2)	0
Total	263	100	279	62	541	162

Legend: NHP: Non-human primate. N: number. P: positive.

**Table 2:** Positive YFV *Callithrix sp* in São Paulo city and their respective distances from green areas or woods, 2017–2018.

*Callithrix* ID	District (Region)	Area	Notification	Distance from green areas or woods
1	Anhanguera (N)	WO	Oct 16 2017	0 m
2	Anhanguera (N)	WO	Oct 16 2017	0 m
3	Vila Guilherme (N)	UR	Oct 25 2017	610 m
4	Santo Amaro (S)	UR	Dec 19 2017	540 m
5	Santana (N)	UR	Dec 19 2017	450 m
6	Santo Amaro (S)	UR	Jan 4 2018	634 m
7	Capão Redondo (S)	UGA	Jan 18 2018	0 m
8	Campo Grande (S)	UR	Jan 19 2018	290 m
9	José Bonifácio (E)	UGA	Jan 24 2018	0 m
10	Santo Amaro (S)	UR	Jan 25 2018	50 m
11	Brasilândia (N)	UR	Jan 27 2018	320 m
12	Pq do Carmo (E)	UGA	Jan 28 2018	0 m
13	Campo Grande (S)	UR	Feb 19 2018	220 m
14	Mandaqui (N)	UR (pet)	Feb 28 2018	210 m
15	Marsilac (S)	WO	Feb 28 2018	0 m
16	Campo Grande (S)	UGA	Mar 12 2018	0 m
17	Ipiranga (S)	UR	Mar 14 2018	520 m
18	Santo Amaro (S)	UGA	Apr 16 2018	0 m
19	Perus (N)	UR	Apr 22 2018	80 m
20	Cangaíba (E)	UR	May 25 2018	550 m
21	São Domingos (N)	UR	May 25 2018	40 m
22	Perus (N)	WO	Sep 12 2018	0 m

Legend: S = South, N = North, E = East, WO = wood, UR = urban, UGA = urban green area.

### YFV in mosquitoes

A total of 5591 female mosquito were collected, 2691 (48.1%) from Aedini Tribe or *Sabethes* genus. Results of entomological collections are shown in Table 3. Three mosquito species were more abundant and occurred frequently: *Aedes scapularis* (N = 1674, 29.9%) occurred in 78 collection sites (76.5%), followed by *Aedes albopictus* (N = 798, 14.3%) in 53 (52%) and *Aedes aegypti* (N = 83, 1.5%) in 24 sites(23.5%). These species were also collected in urban dwellings as well as in urban green areas. We also recorded *Psorophora ferox* in urban green areas and *Haemagogus leucocelaenus* in the woods (Table 3).

**Table 3:** Distribution of potential mosquito species vectors of Yellow Fever in the habitats of entomological surveillance (woods, urban green areas and urban dwellings) during epizootics in São Paulo from October 2017 through December 2018.

Species	Wood			Urban Green Areas			Urban Dwellings			Total	
	n	$\stackrel{-}{\mathrm{X}}$(Std)	n coll	n	$\stackrel{-}{\mathrm{X}}$(Std)	n coll	n	$\stackrel{-}{\mathrm{X}}$(Std)	n coll	n	n coll
*Aedes aegypti*	5	0.2 (0.7)	2	46	1.1 (2.2)	14	32	3.8 (5.7)	8	83	24
*Aedes albopictus*	149	5.0 (7.0)	17	623	14.2 (29.8)	33	26	0.8 (1.5)	3	798	53
*Aedes argyrothorax*	1	–	1	–	–	–	–	–	–	1	1
*Aedes scapularis*	746	33.5 (94.3)	26	894	24.2 (50.3)	47	34	1.7 (3.9)	5	1674	78
*Aedes terrens*	11	0 .4 (1 .7)	4	–	–	–	–	–	–	11	4
*Haemagogus janthinomys/capricornii*	1	–	1	–	–	–	–	–	–	1	1
*Haemagogus leucocelaenus*	72	2 .7 (10 .7)	11	–	–	–	–	–	–	72	11
*Psorophora ferox*	28	1 .3 (4 .7)	7	3	0.1 (0.4)	3	-	-	-	31	10
*Sabethes albiprivus*	10	0 .3 (1 .1)	4	–	–	–	–	–	–	10	4
*Sabethes chloropterus*	1	–	1	–	–	–	–	–	–	1	1
*Sabethes purpureus*	7	0 .3 (0 .8)	5	–	–	–	–	–	–	7	5
*Sabethes soperi*	2	–	1	–	–	–	–	–	–	2	1
Total	1033	–	33ª	1566	–	56ª	92	-	13ª	2691	102^b^

Legend: n: number of females, X ®(Std): arithmetic mean of collections by effort and hours in each site (standard deviation), n coll.: number of collections with presence of the species, a: total entomological collections according habitat type, b: total entomological collections.

**Figure 2:**
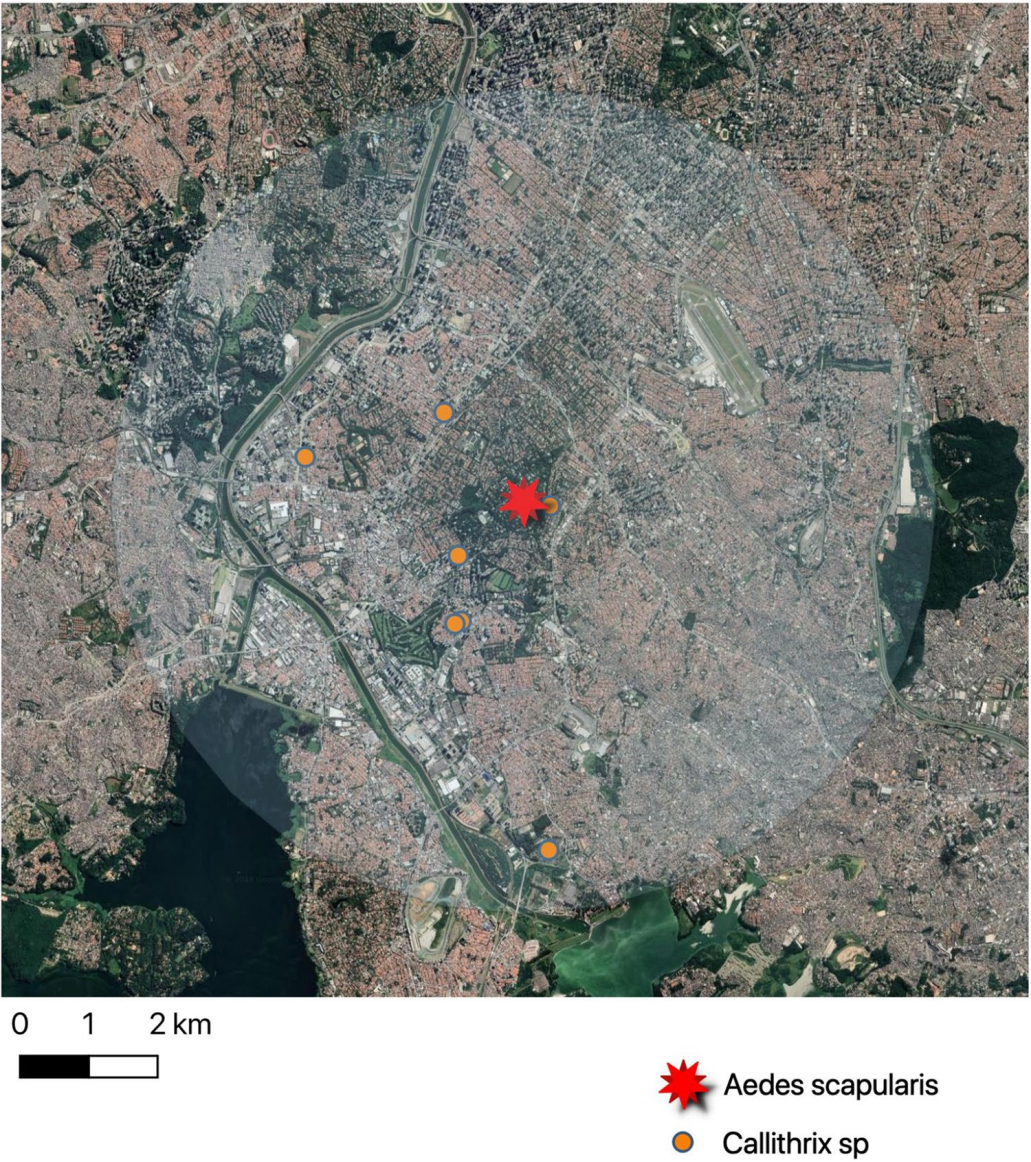
Positive *Callithrix* monkeys and *Aedes scapularis* from Santo Amaro district. The buffer indicates a medium flight range of most Culicidae species (radius = 6Km).

From a total of 437 mosquito pools, 2 from *Haemagogus leucocelaenus* in Horto Florestal (CT values of 19 and 18) collected on December 20th, 2017 and 1 from of *Aedes scapularis*, from the Santo Amaro district collected on February 19th, 2018 (Chácara Flora neighborhood, latitude −23,647,240; longitude −46,683,910), (CT value = 37), were YFV positive. Figure 2 shows the landscape of the Santo Amaro district, where the positive *Aedes scapularis* pool was collected following confirmed epizootic events in the area. YFV was not detected in *Aedes aegypti* or *Aedes scapularis* pools by RT-qPCR. YFV from each of *Haemagogus leucocelaenus* pools was isolated in Vero and C6/36 cells. However, even after 3 passages, YFV was not isolated from the *Ae. scapularis* pool, possible due to low viral load. All positive mosquito pools were tested negative for human RNAse P gene.

The ML phylogenetic tree generated (S1) revealed that sequences belonging to *Alouatta* genus from São Paulo are basal to those of humans, revealing the sylvatic YFV circulation previous to human cases detected in the city.

## Discussion

In 2016, a sylvatic YF outbreak occurred in Minas Gerais State, which spread to other Brazilian states, including São Paulo^18^. Continuous surveillance detected positive cases in humans and NHPs during subsequent years, indicating viral persistence in the Southeast region^19,20^. We now detected positive epizootic events in São Paulo city from October 2017 until September 2018. We describe 18 positive RT-qPCR YFV NHP belonging to the *Callithrix* genus found in urbanized areas within the metropolitan areas of São Paulo city, as well as pools of *Hg. leucocelaenus* from Horto Florestal and one *Ae. scapularis* pool from Santo Amaro. This last district has an area of 15.7 Km^2^ with a density of 38,70 inhabitants/ha, where thousands of people live and circulate daily. Our results show that apparently a unique YFV pattern may have possibly occurred during the 2017–2018 outbreak. Entomological collections executed in these areas did not detect the presence of strictly sylvatic mosquitoes, the main YFV vectors in the southeastern region^21,22^. This situation deserves attention since it may represent a new YF epidemiological pattern, since the circulation of YFV occurred in wooded neighborhoods within metropolitan São Paulo and close to highly populated areas. In addition, one positive *Callithrix* was an indoor pet with presumably no outside exposure.

In Brazil, several species of *Callithrix* monkeys (marmosets) are found, such as *Callithrix jaccus, Cx. penicillata, Cx. geoffroyi* and *Cx. aurita*. However, some species were introduced into new areas, and hybrid individuals are now present in the South and Southeastern regions; their susceptibility to infectious disease is poorly understood. In São Paulo, marmosets are found in city centers close to humans, and sometimes are even used as pets. However, unlike *Alouatta* monkeys*,* which are highly susceptible to YFV^23,24^, marmosets may not be efficient YFV amplifier hosts due to low viral loads, as previously reported^25,26^. As our results demonstrate, *Alouatta* monkeys were restricted to forest areas, mainly in Atlantic Forest preserved areas, while most marmosets were found in squares and other green areas, in close proximity to humans.

Interestingly, entomological collections performed in the city at sites where epizootic events were recorded showed a high frequency of the synanthropic mosquitoes *Aedes scapularis* and *Aedes albopictus.* Although we did not find *Ae. albopictus* and *Ae. aegypti* naturally infected with YFV in our collections, Brazilian populations of these species showed to be competent to transmit this virus ^27^.

According to a previous study that collected Culicidae in the parks of São Paulo during 2010–2011, *Culex quinquefasciatus* was the most common species collected^28^. The genus *Aedes* was represented mainly by *Ae. (Och) fluviatilis and Ae. (Ste) albopictus*, while *Aedes scapularis* were frequent in some parks^24^. However, information regarding mosquitoes diversity are scarce in São Paulo city, and more studies must be done to clarify the real frequency of Culicidae. In the present study, we hypothesize that *Ae. scapularis* mosquitoes could have played a role for YFV transmission in some green areas of São Paulo. This was the most frequent species identified in urbanized areas where YF positive NHP were found. Moreover, we detected one RT-qPCR positive pool in the Santo Amaro district, where 7 marmosets were positive from December 2017 until April 2018, while no *Haemagogus* or *Sabethes* were collected in this district. This pool was also negative for the endogenous primate control (RNAse P), showing the absence of recent human or NHP blood. *Ae. scapularis* is widely distributed in the Americas, and bites preferably in twilight at ground level in fragmented forests, open fields and forest edges^29^. This species shows a synanthropic tendency, occurring in modified environments and breeding in artificial containers^30,31^. It is an anthropophilic opportunistic mosquito that feeds on birds, humans, and NHP^32^. Other entomological surveys have demonstrated YFV-positive pools in Rio de Janeiro and Bahia^15^. More recently, one YFV-positive pool of *Ae. scapularis* was found in Urupês city, São Paulo State, at a site where epizootic cases were absent^33^.

The presence of YFV within an urban area is intriguing. According to recent reports, the movement rate of the current YFV lineage is on average 0.84 (0.50–2.19) km/day^16^ to 4.25 km/day^8^ within the sylvatic cycle, reflecting NHP movements. However, as our results show, during the epidemic period (i.e. January 2018), 7 marmosets and 1 free-living *Alouatta* from the city zoo were YFV positive in distant areas of São Paulo (East, North and South zones), with no forest areas connecting them. According to^34^, NHPs are not responsible for the rapid spread of YFV^30^. Conversely, infected mosquitoes and humans can disperse the virus over great distances^7,35^. Hypotheses concerning YFV dissemination during the 2016–2018 Southeast outbreak were (1) augmentation of conservation areas, (2) high population density of non-immune NHP in the coastal zone, and (3) increased occupation of modified environments by adapted marmosets in *Haemagogus* infested woods^36^. Arboviruses may also be dispersed by windblown infected mosquitoes, as previously demonstrated for Japanese Encephalitis virus in Australia^37^. Nevertheless, this dissemination was likely caused by intense monsoon winds, which is not observed in Brazil. Therefore, considering both the distance and sequence of epizootic events, we may consider the possibility of viral introduction by asymptomatic viremic people, as previously hypothesized^5^, which is not consistent with the classical sylvatic cycle. The spread of the virus with a pattern distinct from the classical sylvatic cycle in an ecological transition zone has also been described in Rio de Janeiro^36^ . It is also important to note that São Paulo city was not a vaccine-recommended area until recently, and most of the 12 million inhabitants were YF naïve before the outbreak. The occurrence of positive NHP and mosquitoes in areas with a susceptible population poses a serious threat for re-urbanization of the disease, and vaccination campaigns must be continued.

Limitations of our study must be acknowledged. We did not conduct experiments about vector competence, since a biosafety level 3 facility was not available, and we did not perform comparative viral sequencing in the mosquitoes and marmosets due to high Ct values. Experiments regarding vector competence of *Ae. scapularis* mosquitoes using this modern YFV lineage are needed, since the detection of the YFV genome in a mosquito does not necessarily mean that the insect can play a role as vector.

In conclusion, YFV was detected in the São Paulo urban area, affecting mainly resident marmosets, with synanthropic *Aedes* mosquitoes probably implicated in local viral transmission. This YF pattern is not consistent with the classic Brazilian cycles, urban or sylvatic, and it suggests a new scenario, probably due to anthropic action and modifications in natural landscapes and callitrichid behavior. Moreover, YFV surveillance based on analysis of deceased NHP has triggered rapid mass vaccination in São Paulo city to prevent human infection, and no human urban case was detected. This epidemiological pattern in a transition area between green and urban areas, with high population density, indicates the need for further studies to subsidize surveillance in order to prevent the re-urbanization of the disease in Brazil.

## Supplementary information

Supplementary Information.
