# Supplementary material for: Possible non-sylvatic transmission of yellow fever between non-human primates in São Paulo city, Brazil, 2017–2018
Source: Sci Rep. 2020 Sep 25;10:15751. doi: 10.1038/s41598-020-72794-x (PMC7519641; doi:10.1038/s41598-020-72794-x)
Supplement: Supplementary file 1 — Supplementary Information. [file 41598_2020_72794_MOESM1_ESM.docx]

Supplementary material

Title: Possible non-sylvatic transmission of yellow fever between non-human primates in São Paulo city, Brazil, 2017-2018

Mariana Sequetin Cunha^1§^, Rosa Maria Tubaki^2,^, Regiane Maria Tironi de Menezes^2^, Mariza Pereira^3^, Giovana Santos Caleiro^1,4^, Esmenia Coelho^3^, Leila del Castillo Saad^5^, Natalia Coelho Couto de Azevedo Fernandes^6^, Juliana Mariotti Guerra^6^, Juliana Silva Nogueira^1^, Juliana Laurito Summa^7^, Amanda Aparecida Cardoso Coimbra^7^, Ticiana Zwarg^7^, Steven S. Witkin^4,8^, Luís Filipe Mucci^3^, Maria do Carmo Sampaio Tavares Timenetsky^9^, Ester Cerdeira Sabino^4^, Juliana Telles de Deus^3^.

1. Vector-borne Diseases Laboratory, Adolfo Lutz Institute, Sao Paulo, Brazil

2. Laboratory of Medical Entomology, Superintendence of Control of Endemic Diseases (SUCEN), Sao Paulo, Brazil

3. Yellow Fever Technical Group, Superintendence of Control of Endemic Diseases (SUCEN), Sao Paulo, Brazil

4. Laboratory of Virology, Institute of Tropical Medicine, University of São Paulo, Sao Paulo, Brazil

5. Divisao de Zoonoses, Centro de Vigilância Epidemiológica, Sao Paulo, Brazil

6. Pathology Center, Adolfo Lutz Institute, Sao Paulo, Brazil

7. Technical Division of Veterinary Medicine and Wildlife, Sao Paulo, Brazil

8. Department of Obstetrics and Gynecology, Weill Cornell Medicine, New York, NY 10065, USA

9. Center of Virology, Adolfo Lutz Institute, Sao Paulo, Brazil

^§^corresponding author

S1

Phylogenetic tree of the 2016-2018 YFV outbreak in southeastern, Brazil. Sequences of YF whole genomes were aligned using MAFTT V.7 (https://mafft.cbrc.jp/alignment/software/). The best model was obtained using MEGA 7 software^38^. The maximum likelihood tree was constructed using PhyML 3.0: new algorithms, methods and utilities (<http://www.atgc-montpellier.fr/>), according to^17^ using a TN93 substitution model with gamma distribution and Fast likelihood-based method ALRT-Sh like. The tree contains sequences accession number, host, local and year. Purple sequences belong to human fatal cases from São Paulo, SP, in 2018. Tree was constructed using FigTree v.1.4.3 with an automatic scale. Blue, pink and green clades corresponds to YFV detected in southeastern Brazil in 2016-2017 (MG=Minas Gerais, ES=Espírito Santo, SP=São Paulo). Scale in nucleotide substitutions per site.


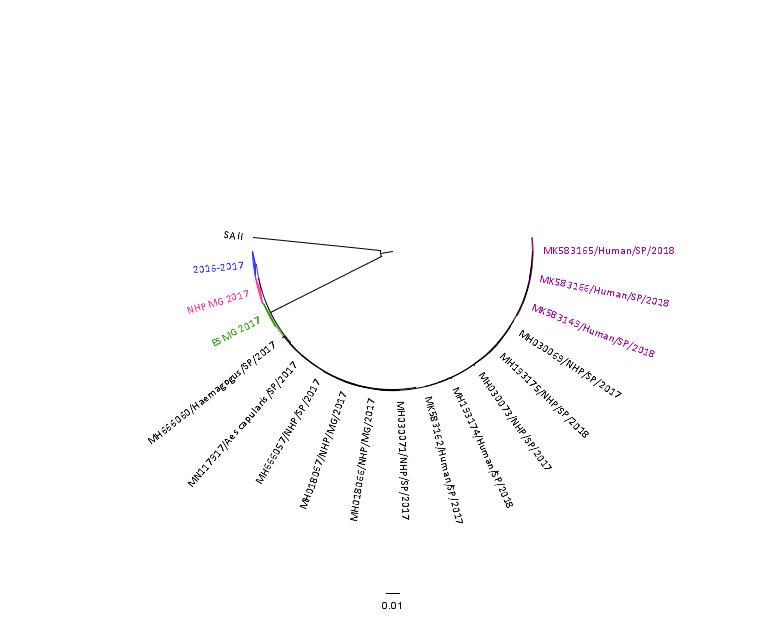


S2

Table containing Genbank accession numbers, year, state and host of YFV SA genotype I samples used for phylogenetic studies. SP=São Paulo, MG=Minas Gerais, ES=Espírito Santo, RJ=Rio de Janeiro. YFV accession number MF004382 (SA II) was used as outgroup.

| GenBank accesion number | Year | State | Host |
| --- | --- | --- | --- |
| MT497523 | 2016 | SP | Alouatta sp |
| MH666056 | 2016 | SP | Alouatta sp |
| MN117917 | 2017 | SP | Aedes scapularis |
| MH018102 | 2017 | ES | NHP |
| MH484429 | 2017 | MG | Callitrix sp |
| MH666060 | 2017 | SP | Haemgagous janthinomys |
| MH666057 | 2017 | SP | Alouatta sp |
| MH018067 | 2017 | MG | Cebidae sp. |
| MH018066 | 2017 | MG | Alouatta sp |
| MH030071 | 2017 | SP | Alouatta sp |
| MK583162 | 2017 | SP | Human |
| MH030073 | 2017 | SP | Alouatta sp |
| MH030069 | 2017 | SP | Alouatta sp |
| MH484431 | 2017 | MG | NHP |
| MH018115 | 2017 | ES | NHP |
| MF170971 | 2017 | MG | NHP |
| MH484430 | 2017 | MG | NHP |
| MH018082 | 2017 | MG | Callithrix sp |
| MH484433 | 2017 | MG | NHP |
| MN117917 | 2017 | SP | Aedes scapularis |
| MH018114 | 2017 | ES | NHP |
| MH018112 | 2017 | RJ | Alouatta sp |
| MH484427 | 2017 | MG | Callithrix sp |
| MH484426 | 2017 | MG | NHP |
| MH018084 | 2017 | MG | Callithrix sp |
| MH018097 | 2017 | MG | NHP |
| MH484432 | 2017 | MG | Alouatta sp |
| MH018106 | 2017 | MG | Alouatta sp |
| MH193174 | 2018 | SP | Human |
| MH193175 | 2018 | SP | Alouatta sp |
| MK583149 | 2018 | SP | Human |
| MK583165 | 2018 | SP | Human |
| MK583166 | 2018 | SP | Human |
